# Relationship between serum ECP and TIgE levels and the risk of postoperative recurrence in patients with chronic rhinosinusitis with nasal polyps

**DOI:** 10.3389/fsurg.2024.1516981

**Published:** 2025-01-13

**Authors:** Qing Zhang, Hui Li, Di Xie, SiJian Fan

**Affiliations:** ^1^ Department of Otolaryngology, YongKang Maternity and Child Health Hospital, Jinhua, Zhejiang, China; ^2^ Department of Otolaryngology, Pei County People’s Hospital, Xuzhou, Jiangsu, China

**Keywords:** chronic rhinosinusitis with nasal polyps, eosinophil cationic protein, total immunoglobulin E, endoscopic sinus surgery, recurrence risk

## Abstract

**Objective:**

This study was undertaken to assess the association between the likelihood of surgical recurrence and serum ECP and TIgE levels in chronic rhinosinusitis with nasal polyps (CRSwNP).

**Methodology:**

Clinical information was gathered retrospectively from 166 cases of surgically treated CRSwNP as well as 60 cases of chronic rhinosinusitis without nasal polyps (CRSsNP). A comparative analysis on serum levels of total immunoglobulin E (TIgE) and eosinophil cationic protein (ECP) was carried out between the two groups. The CRSwNP patients were assigned into recurrence and non-recurrence groups based on the absence/presence of disease recurrence after a 2-year follow-up. An analysis was conducted on the correlation between the patients' clinical data and their serum ECP and TIgE levels. Receiver operating characteristic (ROC) curves were utilized to assess the clinical utility of these two biomarkers.

**Results:**

The CRSwNP participants had higher serum levels of ECP and TIgE (4.28 ± 0.81 > 3.58 ± 0.77 ng/L, *P* < 0.001; 52.99 ± 8.62 > 15.65 ± 3.25 KU/L, *P* < 0.001) compared to CRSsNP participants. Univariate analysis indicated that neutrophil ratio, lymphocyte ratio, Lund-Kennedy score, Lund-Mackay score, SNOT-22 score, olfactory function score, and postoperative recurrence were significantly correlated with serum ECP and TIgE levels. Higher serum levels of TIgE and ECP (4.89 ± 0.79 < 4.11 ± 0.72, *P* < 0.001; 58.74 ± 8.27 < 51.40 ± 8.04, *P* < 0.001) were detected in the recurrence groups vs. the non-recurrence group. Multivariate analysis showed that serum ECP and TIgE were independent risk factors for recurrence of CRSwNP. Serum ECP and TIgE levels were found to be predictive of postoperative recurrence risk in CRSwNP patients (AUC: 0.77, 0.74, 0.84; *P* < 0.05) according to ROC curve analysis. Significant differences were not observed in any general clinical data.

**Conclusion:**

The findings suggest that elevated serum ECP and TIgE levels in patients with CRSwNP can be as good predictors for the risk of recurrence after endoscopic sinus surgery.

## Introduction

Chronic rhinosinusitis (CRS) represents a common inflammatory condition of the sinuses and nasal cavity, affecting approximately 12% of the general population (1). CRS with nasal polyps (CRSwNP) and CRS without nasal polyps (CRSsNP) are two primary phenotypic subtypes. CRSwNP is characterized by the formation of single or multiple nasal polyps resulting from prolonged inflammation and hyperplasia of the nasal and sinus mucosa (2). Symptoms typically include nasal obstruction, purulent nasal discharge, facial pressure or pain, and hyposmia lasting for more than 12 weeks (3). CRSwNP not only affects patients’ quality of life but can also trigger severe complications such as nasal blockage, impaired olfactory function, and breathing difficulties (4, 5). The most extensively applied therapy for CRSwNP is endoscopic sinus surgery (ESS) (6, 7). However, postoperative recurrence is frequently observed, with reported recurrence rates ranging from 5% to 60%, and even up to 80% after a 12-year follow-up (8). This recurrence increases medical visits and prolongs the disease course, causing significant negative effects on the physical and psychological status of patients (9).

Accurately forecasting the risk of postoperative recurrence in CRSwNP patients and developing more targeted therapeutic plans is a major concern in the medical community. Previous studies have suggested the utility of factors such as allergic reactions, asthma, history of ESS, inflammatory cells, and related cytokines to predict postoperative recurrence in CRSwNP patients (10, 11). Other researchers have unveiled that eosinophil levels are also strongly linked to postoperative recurrence (11). The pathophysiological characteristics of CRSwNP are complicated and varied, involving diverse cells and molecular mechanisms of the immune system. Among numerous inflammation-related biomarkers, eosinophil cationic protein (ECP) and total immunoglobulin E (TIgE) have garnered widespread attention due to their prominent roles in the context of allergic reactions and inflammatory processes (12). ECP, a potent toxic protein derived from eosinophils, exerts antibacterial, antiviral, and immunoregulatory functions (13). Elevated ECP levels typically indicate eosinophil activation and the extent of tissue inflammation. In CRSwNP patients, increased ECP levels are often relevant to the severity of the disease and the risk of recurrence (14). Another marker IgE is a crucial antibody for allergic reactions that can mediate the degranulation of mast cells and basophils, contributing to the release of histamine and other inflammatory mediators, ultimately triggering allergic symptoms (15). Elevated IgE levels are typically related to allergic diseases and inflammatory states (16). However, most studies only reported IgE levels in nasal polyp tissues (17, 18) or specific IgE (sIgE) levels (18, 19). In this regard, validating serum ECP and TIgE levels in CRSwNP patients and excavating their relationship with postoperative recurrence risk is of great concern for understanding the disease's pathogenesis and developing personalized therapeutic strategies.

Herein, we probe into the link between serum ECP and TIgE levels in CRSwNP patients and the risk of postoperative recurrence. By testing serum samples from patients and conducting follow-ups, we analyze the changes in ECP and TIgE levels and their correlation with recurrence. The goal is to provide reliable biomarkers for clinical practice, assist physicians in developing more precise and customized therapeutic regimens, reduce postoperative recurrence rates, and improve the quality of life for patients.

## Methods

This retrospective study aims to evaluate the impact of serum ECP and TIgE levels on the risk of recurrence after ESS. The study population included 166 CRSwNP patients and 60 CRSsWP patients from our hospital between September 2020 and September 2021. The inclusion criteria for patients were: (a) met the diagnostic criteria of the “European Position Paper on Rhinosinusitis and Nasal Polyps 2020”; (b) aged ≥18 years; (c) no relevant medication treatment (including corticosteroids, antibiotics, or antihistamines) within 4 weeks before surgery. The exclusion criteria were: (i) fungal sinusitis; (ii) antrochoanal polyps; (iii) history of ESS; (iv) autoimmune diseases (based on previous systematic detection); (v) concomitant or previous biological therapy and intranasal steroid therapy within the last year before enrolment; (vi) history of aspirin or NSAID intolerance.

First of all, general data were gained from two groups of subjects, encompassing gender, age, history of asthma, history of prior surgery, history of smoking, Lund-Kennedy score, Lund-Mackay score, SNOT-22 score, and olfactory function score. 4 ml of fasting elbow venous blood was taken from the patient, centrifuged at 3,000 rpm at low temperature for 5 min, and the supernatant was separated. Then, serum ECP and TIgE levels were detected by enzyme-linked immunosorbent assay. The olfactory function of patients before surgery was evaluated by the five-taste test olfactory solution (20). The 5 odors were configured with 8 different concentrations from low to high, and each concentration score was −2, −1, 0, 1, 2, 3, 4 and 5 points. The test paper dipped in olfactory solution was placed 1–2 cm in front of the nasal vestibule of the patient for identification. The average olfactory threshold value (total fraction of 5 odors and divided by 5) was calculated. The average olfactory threshold value was divided into 5 levels: ≤1 was divided into 1 level, indicating normal olfactory sense; A score of >1.0–2.5 is grade 2, indicating mild olfactory disturbance; >2.5–4.0 is divided into 3 levels, indicating moderate impairment of olfactory function; >4.0–5.5 is divided into 4 grades, indicating severe impairment of olfactory function; >5.5 is graded as 5, indicating loss of olfactory function. Based on the median serum ECP and TIgE levels in the CRSwNP group, these patients were categorized into high-expression and low-expression groups to analyze the correlations of between serum ECP and TIgE levels with clinical data. Additionally, the CRSwNP patients were sub-divided into non-recurrence and recurrence groups based on postoperative outcomes, to investigate whether serum ECP and TIgE levels potentially correlated with the risk of postoperative recurrence.

A single-factor analysis was employed to assess the relationships between serum ECP and TIgE levels as inflammatory markers and the clinical data of the patients. Univariate and multivariate analyses were used to explore the risk factors for recurrence of CRSwNP patients. Receiver operating characteristic (ROC) curve analysis was utilized to evaluate the clinical performance of these inflammatory markers in predicting postoperative recurrence risk. Statistical analyses were conducted using SPSS 20.0 (IBM Corp., Armonk, NY, USA). Normally distributed continuous data are described as mean ± standard deviation, and comparisons between two samples were performed using the *t*-test. Non-normally distributed continuous data are presented as medians (minimum, maximum), and comparisons between groups were analyzed using the Mann-Whitney *U* test. Categorical data are displayed as [*n* (%)] and were compared using the chi-square test. Statistical significance was assumed if *P* < 0.05.

## Results

The general characteristics of the CRSwNP patient group and the CRSsWP group summarized in Table 1 were compared. Age, gender, history of asthma, previous operation history, and history of smoking did not noticeably differ between the two groups (*P* > 0.05). However, prominent differences were observed concerning the Lund-Kennedy, Lund-Mackay, SNOT-22, and olfactory function scores (*P* < 0.05).

**Table 1:** Analysis of general date of patients.

Factor	CRSsWP	CRSwNP	*Z*/*χ*^2^/*t*	*P*
Age	42.5 (31, 53)	43.5 (29, 58)	1.58	0.11
Gender (Male/female)	32/28	87/79	0.02	0.90
History of asthma (Yes/No)	0/60	9/158	3.00	0.08
Previous surgery (Yes/No)	0/60	10/156	3.78	0.05
Lund-Kenendy	8.85 ± 3.34	10.61 ± 3.68	18.14	0.001
Lund-Mackey	14 (13, 17)	17.5 (8, 23)	6.413	<0.001
SNOT-22	31.83 ± 5.68	36.24 ± 13.85	12.30	0.018
Smoking history (Yes/No)	15/45	24/142	3.43	0.06
Olfactory function score	2.0 (−2, 3)	3.0 (−1, 5)	7.293	<0.001

CRSwNP, chronic rhinosinusitis with nasal polyps; CRSsWP, chronic rhinosinusitis without nasal polyps.

We also compared the serum levels of ECP and TIgE between the two groups. As depicted in Table 2, higher levels of serum TIgE and ECP were noted in the CRSwNP group than in the control group (TIgE: 4.28 ± 0.81 > 3.98 ± 0.77 ng/L; ECP: 52.99 ± 8.62 > 50.30 ± 8.11 KU/L).

**Table 2:** Comparison of serum ECP and TIgE levels between the two groups (CRSwNP group and CRSsWP group).

Factor	ECP (ng/L)	TIgE (KU/L)
CRSwNP	4.28 ± 0.81	52.99 ± 8.62
CRSsWP	3.98 ± 0.77	50.30 ± 8.11
*t*	2.490	2.104
*P*	0.014	0.037

Additionally, the correlations between clinical data and serum levels of ECP and TIgE in patients are present in Table 3. Age, gender, history of asthma, previous operation history, and smoking history did not significantly affect serum ECP and TIgE levels in CRSwNP patients (*P* > 0.05). Neutrophils, lymphocytes, Lund-Kennedy scores, Lund-Mackay scores, SNOT-22 scores, olfactory function scores, and postoperative recurrence, however, exhibited significant variations (*P* < 0.05). In contrast to the non-recurrence group, the recurrence group displayed elevations in serum levels of ECP and TIgE (ECP: 4.89 ± 0.79 > 4.11 ± 0.72 ng/L; TIgE: 58.74 ± 8.27 > 51.40 ± 8.04 KU/L, Table 3).

**Table 3:** Univariate analysis of patients’ serum ECP and TIgE levels and correlation with clinical data of the patients.

Factor		ECP	*t*	*P*	TIgE	*t*	*P*
Age	Age < 42	4.24 ± 0.67	0.49	0.63	53.78 ± 8.30	0.34	0.18
	Age ≥ 42	4.30 ± 0.83			51.99 ± 8.96		
Gender	Male	4.32 ± 0.83	0.61	0.54	53.06 ± 9.96	0.11	0.91
	Female	4.24 ± 0.78			52.91 ± 6.91		
History of asthma	Yes	4.50 ± 0.92	0.84	0.40	49.12 ± 9.75	1.39	0.17
	No	4.23 ± 0.80			53.21 ± 8.53		
Previous surgery	Yes	4.56 ± 0.87	1.06	0.29	52.08 ± 8.98	0.03	0.97
	No	4.26 ± 0.80			52.99 ± 8.63		
Neutrophil (%)	>4.2	4.47 ± 0.87	2.94	0.004	54.92 ± 7.96	2.78	0.006
	≤4.2	4.11 ± 0.71			51.28 ± 8.86		
Leukomonocyte (%)	>2	4.52 ± 0.74	3.69	<0.001	54.81 ± 8.69	2.61	0.01
	≤2	4.07 ± 0.80			51.38 ± 8.27		
Lund-Kenendy score	>11	4.62 ± 0.84	4.59	<0.001	56.25 ± 8.70	4.09	<0.001
	≤11	4.06 ± 0.71			50.89 ± 7.93		
Lund-Mackey score	>17.5	4.50 ± 0.84	3.57	<0.001	55.49 ± 8.23	3.89	<0.001
	≤17.5	4.06 ± 0.71			50.49 ± 8.31		
SNOT-22 score	>36	4.56 ± 0.87	4.58	<0.001	55.63 ± 8.64	3.98	<0.001
	≤36	4.02 ± 0.64			50.53 ± 7.89		
Smoking history	Yes	4.29 ± 0.87	0.08	0.94	50.17 ± 8.22	1.74	0.08
	No	4.28 ± 0.80			53.47 ± 8.62		
Olfactory function	>3	4.92 ± 0.82	3.72	<0.001	57.75 ± 9.15	2.52	0.01
	≤3	4.20 ± 0.77			52.41 ± 8.40		
Recurrence after surgery	Yes	4.89 ± 0.79	5.64	<0.001	58.74 ± 8.27	4.82	<0.001
	No	4.11 ± 0.72			51.40 ± 8.04		

Univariate analysis of risk factors for disease recurrence in patients with CRSwNP showed that there were no differences in age, gender, history of asthma, previous surgery, Lund-Kenendy score, smoking history and olfactory function between the two groups (*P* > 0.05). However, there were significant differences in neutrophils, lymphocytes and SNOT-22 between the recurrence and non-recurrence groups (*P* < 0.05). See Table 4 for details.

**Table 4:** Univariate analysis of the risk of recurrence in patients with CRSwNP.

Factor		Non-recurrence	Recurrence	*Z*/*χ*^2^/*t*	*P*
Age		43 (29, 58)	44 (35, 53)	0.588	0.557
Gender	Male	68	19	0.002	0.960
	Female	62	17		
History of asthma	Yes	124	33	0.760	0.383
	No	6	3		
Previous surgery	Yes	124	32	2.101	0.147
	No	6	4		
Neutrophil (%)		4.09 ± 0.86	4.52 ± 0.82	2.651	0.009
Leukomonocyte (%)		1.97 ± 0.41	2.23 ± 0.51	3.235	0.001
Lund-Kenendy score		10.33 ± 3.53	11.61 ± 4.07	1.861	0.064
Lund-Mackey score		17 (8, 23)	18 (10, 22)	1.949	0.051
SNOT-22 score		35 (4, 88)	43 (11, 71)	2.478	0.013
Smoking history	Yes	113	29	0.924	0.336
	No	17	7		
Olfactory function		3 (−1, 5)	3 (0, 5)	0.530	0.596

In the recurrence group, compared to the non-recurrence group, there was an increase in serum levels of ECP and TIgE postoperatively (4.89 ± 0.79 > 4.11 ± 0.72 ng/L; 58.74 ± 8.27 > 51.40 ± 8.04 KU/L). Please refer to Table 5 for details.

**Table 5:** Comparison of serum ECP and TIgE levels in the two groups (recurrence and non-recurrence groups).

Factor	Non-recurrence	Recurrence	*t*	*P*
ECP	4.11 ± 0.72	4.89 ± 0.79	5.64	<0.001
TIgE	51.40 ± 8.04	58.74 ± 8.27	4.82	<0.001

Multivariate Logistic regression analysis showed that ECP and TIgE were independent risk factors for recurrence of CRSwNP (*P* < 0.05). See Table 6 for details.

**Table 6:** Multivariate analysis of risk factors for recurrence in patients with CRSwNP.

Factor	*β*	SE	Wald $x^{2}$ value	*P*-value	OR (95% CI)
Constant	14.569	2.564	32.287	0.000	0.000
Neutrophil (%)	0.349	0.271	1.664	0.197	1.418
Leukomonocyte (%)	0.861	0.556	2.396	0.122	2.365
SNOT-22 score	0.009	0.016	0.339	0.561	1.009
ECP	1.018	0.345	8.681	0.003	2.767
TIgE	0.092	0.030	9.208	0.002	1.096

**Figure 1:**
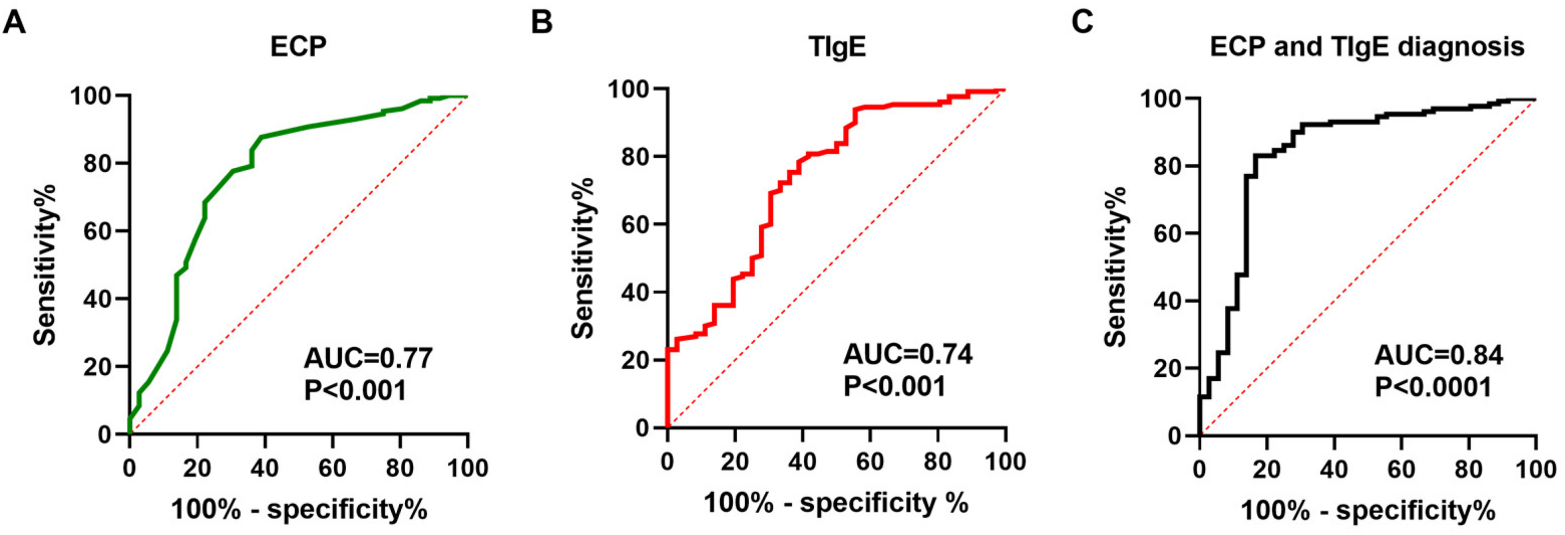
Serum ECP and total IgE levels are capable of predicting the risk of postoperative recurrence in patients with CRSwNP. **(A–C)** ROC curves were generated to evaluate the relationships between serum ECP/total IgE levels and the risk of recurrence in CRSwNP patients.

The ROC curve analysis for predicting postoperative recurrence risk in CRSwNP patients demonstrated a significant predictive value of serum levels of ECP and TIgE (*P* < 0.05). The area under the curve (AUC) values for serum ECP-based, TIgE-based, and both ECP and TIgE-based diagnoses were 0.77, 0.74, and 0.84, respectively. The optimal diagnostic cutoff values were 0.4774 ng/L for ECP alone, 0.3957 KU/L for TIgE alone, and 0.6641 for combined ECP and TIgE. The sensitivity and specificity were 83.85% and 63.89% for ECP alone, 78.46% and 61.11% for TIgE alone, and 83.08% and 83.33% for combined ECP and TIgE, respectively. Please refer to Table 7 and Figure 1A,B for details.

**Table 7:** Value analysis of serum ECP and TIgE levels in predicting the risk of recurrence after ESS.

Factor	AUC	Sensitivity%	Specificity%	Optimum cutoff value
ECP	0.77	83.85%	63.89%	0.4774
TIgE	0.74	78.46%	61.11%	0.3957
ECP and TIgE diagnosis	0.84	83.08%	83.33%	0.6641

## Discussion

This study explored the prediction of postoperative recurrence in patients with CRSwNP (chronic sinusitis with nasal polyps) from a new perspective, focusing on the role of serum ECP and TIgE levels in the prediction of recurrence. While previous studies have focused on local tissue or nasal secretions and multiple biomarkers (such as eosinophils, IL-5, and sIgE), this study is unique in highlighting the use of serum markers, identifying that ECP and TIgE are significantly associated with postoperative recurrence as risk factors, and highlighting the importance of serum markers in predicting recurrence.

CRS is a common clinical condition encountered in otolaryngology practice, with CRSwNP being one of its subtypes. Approximately 20%–30% of all CRS patients are diagnosed with CRSwNP (21). CRSwNP exhibits high disease heterogeneity and involves intricate immunological mechanisms, which can markedly impact the outcomes of surgical and pharmacological treatments (22). Postoperative recurrence is a major concern for many patients, with studies reporting recurrence rates of up to 40% at 18 months post-surgery (23) and 55.6% in Chinese populations within a 2-year follow-up (24). Given the elevated recurrence rates in CRSwNP, early identification of patients prone to recurrence prior to surgical treatment is crucial for selecting appropriate therapeutic interventions and enhancing personalized care.

In this scenario, excavating candidate biomarkers to forecast postoperative recurrence is urgently needed. Seeking precise methods to predict postoperative recurrence is imperative. Researchers have extensively studied a battery of biomarkers, such as eotaxin, IL-17A, and RANTES levels (25), tissue eosinophil counts and percentages (26), macrophages (27), ethmoid/maxillary (E/M) sinus ratio, fraction of exhaled nitric oxide (FeNO), tissue neutrophil count, tissue IL-5 levels, tissue ECP, as well as Charcot-Leyden crystal protein (CLC) or IgE in nasal secretions (18), serum B7-H4 (28), CSF1R, and CSF1 levels (29, 30).

In the pathogenesis of CRSwNP, IgE-mediated activation of type 2 inflammatory cells like mast cells, basophils, and eosinophils is considered central. TIgE serves as a critical indicator of allergic responsiveness in the body, and CRSwNP patients are commonly encountered with allergic disorders. Current research predominantly pays attention to the predictive role of IgE levels in nasal polyp tissue or sIgE levels in CRSwNP recurrence (31) but relatively less attention to TIgE levels in serum. Regarding the role of IgE in the disease, specific targeted therapies have been developed in clinical practice. For instance, Omalizumab strikingly improves endoscopic, clinical, and patient-reported outcomes in severe CRSwNP cases resistant to intranasal corticosteroids, exhibiting good tolerability (32, 33).

ECP is a protein released by eosinophils with cytotoxic and immune-regulatory properties, extensively implicated in inflammatory disorders. ECP contributes to the formation of nasal polyps and the chronic progression of CRS through diverse mechanisms, involving disruption of epithelial cells, stimulation of fibrosis, and aggravation of inflammatory responses. Specifically, ECP may compromise epithelial barrier function, facilitating the invasion of foreign substances and causing tissue damage. Moreover, it can promote local tissue proliferation and structural changes by stimulating fibrotic processes. Additionally, ECP strengthens immune responses to antigens and induces more severe inflammatory reactions. These mechanisms synergistically contribute to the formation of nasal polyps and the sustained progression of CRS. Most research has examined ECP levels in tissues and secretions, indicating that measuring ECP levels could be helpful, especially in eosinophilic CRS (34–36).

This study investigates the expression patterns of serum ECP and TIgE in patients with CRSwNP and their relationships with the risk of recurrence after ESS. Markedly elevated levels of serum ECP and TIgE were detected in the CRSwNP population in comparison to the CRSsNP population. According to the findings of Tsai et al. (37), the CRSwNP patients in the recurrence group had greater serum ECP and TIgE levels than those in the non-recurrence group. Multivariate analysis showed that serum ECP and TIgE were independent risk factors for postoperative recurrence of CRSwNP patients. The analysis of ROC curves demonstrated that serum ECP and TIgE could serve as predictive indicators for postoperative recurrence in patients with CRSwNP. Furthermore, the combined testing approach exhibited superior efficacy (*P* < 0.05) in prognosticating the risk of recurrence. These findings imply that elevations in preoperative serum ECP and TIgE levels are linked to a higher chance of post-ESS recurrence. It is hypothesized that ECP can damage epithelial cells and extracellular matrix components, leading to tissue remodeling and polyp formation. High levels of TIgE indicate a persistent allergic response, where TIgE interacts with high-affinity IgE receptors on mast cells, eosinophils, and basophils, activating these cells to release pro-inflammatory mediators. This consequently exacerbates local inflammation of the nasal and sinus mucosa, expediting polyp growth and recurrence.

The levels of serum ECP and TIgE in CRSwNP patients are highly relevant to multiple clinical parameters. Univariate analysis in this research showed that higher levels of ECP and TIgE were associated with increased neutrophil and lymphocyte counts, higher Lund-Mackay scores, higher Lund-Kennedy scores, higher SNOT-22 scores, impaired olfactory function, and postoperative recurrence. These results suggest that serum ECP and TIgE levels not only reflect the extent of sinus inflammation and polyp burden but also correlate with olfactory dysfunction in CRSwNP patients, indicating their involvement in the mechanisms of sensory loss.

This study has the following limitations. First, because of the retrospective design, we faced constraints in data collection, particularly regarding the lack of comprehensive peripheral blood and tissue eosinophil counts (ECP and TIgE) for all patients. The absence of eosinophil data may have overlooked important aspects of the inflammatory response. The possibility of selection bias also existed. Secondly, TIgE and ECP levels may be disturbed by external factors, such as seasonal allergies, acute infections, and thus have some influence on the results. In addition, the single-center design of this study may limit the generalizability of the results. Future studies should use prospective multicenter cohort studies combined with histopathological analysis to endotype CRSwNP patients, especially to distinguish between eosinophilic and non-eosinophilic subtypes. This will further clarify the predictive value of ECP and TIgE levels in different subtypes and explore their correlation with the immunopathological characteristics of patients. In addition, ECP and TIgE should be considered as potential markers to guide individualized treatment strategies for patients with different subtypes to improve the long-term treatment outcome of patients with CRSwNP.

This study has several limitations. As a retrospective analysis, we faced constraints in data collection, particularly regarding the lack of comprehensive peripheral blood and tissue eosinophil counts (ECP and TIgE) for all patients. This gap limits our ability to fully assess the role of these biomarkers in surgical outcomes. While we focused on serum inflammatory cytokines, which are more accessible, the absence of eosinophil data may have overlooked important aspects of the inflammatory response. Future prospective studies should aim to systematically collect detailed eosinophil counts to better understand their relationship with surgical outcomes.

Preoperative detection of serum ECP and TIgE levels can be used to evaluate the condition of patients with CRSwNP. Identification of patients with high levels of these markers is helpful for stratification of postoperative recurrence risk and treatment planning. Patients with high ECP and TIgE levels may benefit from adjuvant medical therapy to reduce eosinophilic inflammation and allergic reactions. Biological therapies targeting eosinophil activation and IgE production, such as anti-IL-5 or anti-ige antibodies, can be considered for high-risk patients. Intensive postoperative follow-up and early intervention can improve the long-term prognosis of patients.

## Conclusion

Conclusively, this investigation addresses the critical role that pre-operative detection of serum TIgE and ECP levels can forecast postoperative recurrence in patients with CRSwNP. Following ESS, there is a greater chance of polyp regrowth and symptom recurrence when these biomarkers are elevated. With the utilization of these biomarkers in the preoperative evaluation of CRSwNP, illness management may be more accurately performed, individualized treatment plans can be more easily created, and patient outcomes can be improved.

## Data Availability

The original contributions presented in the study are included in the article/Supplementary Material, further inquiries can be directed to the corresponding author.
